# Professional Longings*This article, written while an invalid, was sent to the “Dental Cosmos,” was rejected by the editor, and *kindly* returned by the Publisher. By way of appeal, it was read to the “Mad-river Valley Society,” and the members, at the time ignorant of its history, referred it to the Publication Committee, with the request that it be forwarded to the “Dental Register.” The writer is not responsible for its appearance in print, but has no fault to find either with its rejection or publication.

**Published:** 1866-11

**Authors:** Geo. Watt


### DENTAL REGISTER.

VOL. XX.] NOVEMBER, 1866. [NO. 11.

### Original Communications.

### PROFESSIONAL LONGINGS.*

BY GEO. WATT.

Read at a meeting of the Mad-river Valley Dental Society.

DURING the Irish famine, Henry Russell was singing,

"There's a good time coming."

And he was a true philosopher who responded from the crowd, "Could ye fix the date, Mr. Russell?" Aye, the date! That is it that troubles us all.

" Hope, like the glimmering taper's light,
Illumes and cheers the way;
And still, as darker grows the night,
Emits a brighter ray."

But, if we only knew *when* that light is to brighten and dazzle till the darkness flies away—if we knew just the time

* This article, written while an invalid, was sent to the "Dental Cosmos," was rejected by the editor, and *kindly* returned by the PUBLISHER. By way of appeal, it was read to the "Mad-river Valley Society," and the members, at the time ignorant of its history, referred it to the Publication Committee, with the request that it be forwarded to the "Dental Register." The writer is not responsible for its appearance in print, but has no fault to find either with its rejection or publication.

that hope is to become fruition, how much of our care and anxiety for the future would be dispelled!

But if this train of thought is to be followed out, the heading of this article is a misnomer; and *human* longings is its appropriate title. But this would be a range too wide for my pen, and too general for a journal devoted to a specialty. So, let the hopes, the cares, the cravings and desires of our special profession claim our present attention.

"There is a natural body, and there is a spiritual body," we are told by the learned Apostle of the Gentiles. This statement is usually held as if it read, "There *is* a natural, and there *will be* a spiritual body." But, in the obvious meaning of the language, the spiritual body has its existence at the time of writing, as really, and in the same sense, as the natural body. The "body, soul and spirit" are usually spoken of by inspiration as so many separate, special realities; and why the popular mind recognizes the separate identity of the body, while it denies such identity to the other two, is hard to understand.

Our Creator has given us two sources of information concerning Himself and His works. Leaving the critic to object to our terms, if he pleases, let us call one the Book of Nature and the other the Book of Revelation. These two volumes do not, to our limited capacities, teach the same things; nor, where they do coincide, do they teach to the same extent, or with equal clearness. But that they never disagree is plain, in that both are emanations from Him who is "the same yesterday, to-day and forever."

Then does the *first volume* teach anything about the *existence*, nature, capacities, or powers of the spiritual body? Let us read. What presumption! Let a blind idiot translate the Egyptian hieroglyphics; but let not man, in this benighted state, attempt more than a few letters of the alphabet of this wonderful book!

And let us learn the first letter from a fellow-creature, innocent, humble and happy. If a crab loses one of its members, it is well known that a new one grows in its place. It grows the right size, and of a proper shape. The nutritive powers of the animal are not allowed to act at random. They are controlled by an agency unseen by material eyes. Something tells them where to lay each particle of matter, and when they have brought material enough. Some principle, or *thing*, acts as the Creator's vicegerent, and nothing is bungled. The animal grew to its normal shape and size, in the first place, under the control of this agency, and now it controls the repairs. Is here a hint of a spiritual influence? "There is a spiritual body."

A hint, not less plain, is given by the reparative process in higher orders of animals. Wounds of the soft parts in man are healed, form and function being mainly restored; and even bony tissues are reproduced, so as to demonstrate that some unseen power is trying to retain to man his normal shape and condition. And who does not know that when a part of a member is severed from the body, that member remains unconscious of its loss, and that the news of its severance is conveyed to the soul, or to the individual's consciousness, not by itself, but by other organs? When the roll of the company is called, after a battle, every man that answers to his name is regarded and reported as alive and present. When the soul (or the brain, may be), as captain of the organization, calls the roll of the bodily members, the dissevered member answers as it always did. The man who has lost an arm feels as if his arm and hand were still present. Irritation of a nerve fiber is felt, not at the end of the stump, but at the ends of the fingers which have been removed. That is, the feeling is just the same as if the fingers were present. They promptly answer "*Here!*" to the roll-call; and who, in our present state of knowledge, has a right to report them absent? Are there spiritual fingers to the spiritual body? "There is a spiritual body."

What is true of the fingers is true of all the organs. It is on this principle that "tooth-edgedness" and toothache are felt in artificial teeth—are felt by those who have no teeth. The organs, which, it is claimed, have been removed, or their spiritual prototypes, are simply answering to roll-call.

Do these hints indicate that the "natural body" (that body formed by an aggregation of the physical organs) is simply the servant, or the instrument of the spiritual body? Does it not appear that the spiritual body fashions, moulds or shapes the physical organs, the "natural body," to suit its own ends? That the physical, or natural, body, is not essential to, and, indeed, has nothing to do with personal identity, is evidenced from a single consideration. Those of us who have reached middle age do not possess the smallest particle of the physical bodies of our childhood; yet it is evident to our consciousness that we are the same persons. Matter which then constituted parts of our natural bodies, for aught we know, now aids in forming the bodies of beasts, birds or reptiles; but in this whole process of waste and repair there is not the least confusion of identity, for the soul and the spiritual body are not modified by such things, but remain the same.

That the spiritual body is not obvious to our senses, is no objection to the theory of its existence; for spirits are all around us, ministering to our spiritual wants, or tempting us, through the instrumentality of our passions and propensities, to deeds of crime. Even the aged spirit who claimed that he was "going to and fro in the earth, and walking up and down in it," and who is described, by a higher authority, as one who "goeth about like a roaring lion," is not recognized by our ordinary consciousness. For a spirit, or a spiritual body, to be invisible, it needs simply to be transparent. But who, Oh, who! will satisfy our longing desires, by telling us what a spirit is, and how it acts on, and what its relation to matter is, and how it communicates with a kindred spirit?

When these questions are answered, and these longings satisfied, then we will know something of physiology. But here we know only in part; but there is a place, and there will be a time, where and when we will know even as we are known.

"One of the joys of our heaven shall be," I doubt not, a fuller and clearer knowledge of the refining and ennobling sciences than it is possible for us to obtain here. The existence of these longings is an evidence that they are to be gratified; and the limited attainments we are able to make now are an earnest of that which is in store for a higher and better state of existence.

Turn which way we will, in pursuit of professional knowledge, our view is obstructed by the mists and fogs of doubt and uncertainty. Take, for example, the disease we encounter most frequently. It goes on in its work of destruction, torturing childhood, turning the smile of the blushing maiden to a ghostly grin, bringing on premature old age, and shortening human life; and yet we know almost nothing of its nature, essence, or causes, and confess to this ignorance, by continuing to give it a name which we know is a misnomer.

When a dentist looks at a decayed tooth, he ought to be able, from its appearance, and from accompanying circumstances, to tell exactly what chemical agent induces the decay. He should know, also, the composition, the source or origin, and the *modus operandi* of the agent, as well as its antidote, and the proper mode of preventing its formation. But who is to tell us these things, and when will he tell us? And why must little, unweaned babes suffer the untold tortures of toothache? And why need they suffer the ordinary agonies of first dentition? Must the nutritive functions always be so perverted, that this process, which ought to be a blessing as painless as sleep, shall prove a prolific source of misery and death? Who will teach us the laws of life and health, so clearly and thoroughly as to enable us to rear children who, with painless dentition, shall develop dental organs that will last to old age?

"Prophets and kings desired it long,
But died without the sight."

And so, I fear, many, very many, of us who have desired and longed to see these blessings rest on our race, will die with our longing souls unsatisfied. But what of it? Abraham *rejoiced* to see the day of Christ; "and he saw it and was glad." The best view of the promised land ever obtained by the eye of an Israelite was that of Moses from the top of Nebo. So now, those in the profession whose faith and hope are normally developed and properly cultivated, have a Pisgah vision of the "good time" that is to bless mankind, through the instrumentality of our profession; and we see it and are glad.

What a wide field is still open for professional exploration, and how much wider the space covered by our ignorance than by our knowledge! Yet how often do we find men in our profession who think, no doubt, we are the people, and wisdom will die with us! As a conductor of a professional periodical, I have often requested brethren to write something for publication; and the almost invariable answer has been, "Oh, there's nothing left to write about." I have suggested to individuals to take some subject and give it a special investigation, and then give us the results of their examinations; and the answers received have been, in substance, that there is no subject but has been examined, and written to death. Now, if any suggestion here should prove useful in exciting to study, in stimulating our profession to explore untrodden ways, I will be amply rewarded for my trouble in writing this.

